# Adenosquamous carcinoma of the breast: a population-based study

**DOI:** 10.1007/s12282-021-01222-3

**Published:** 2021-02-12

**Authors:** Cheng Xu, Zhangyuan Gu, Juan Liu, Xiaoyan Lin, Cheng Wang, Jiejing Li, Yun Fu, Xiaolin Cheng, Zhigang Zhuang

**Affiliations:** 1 Department of Breast Surgery, Yangpu Hospital, Tongji University School of Medicine, Shanghai, 200090 China; 2 Department of Breast Surgery, Shanghai First Maternity and Infant Hospital, Tongji University School of Medicine, Shanghai, 200040 China; 3 Department of Breast Surgery, Huangpu Branch, Shanghai Ninth People’s Hospital, Affiliated To Shanghai, Jiao Tong University School of Medicine, Shanghai, China

**Keywords:** Breast neoplasms, Carcinoma, Adenosquamous, Carcinoma, Ductal, Breast, Carcinoma, Squamous cell, Prognosis

## Abstract

**Background:**

To summarize the clinicopathological characteristics, prognosis, and management of breast adenosquamous carcinoma (ASC).

**Methods:**

A population-based study was performed using retrospectively extracted data from the Surveillance, Epidemiology, and End Results database for breast cancer patients with histological diagnoses of ASC, infiltrating duct carcinoma (IDC) and squamous cell carcinoma (SCC) from 2004 to 2016.

**Results:**

ASC presented similar tumor size but low histological grade and less lymph node metastasis compared to IDC. ASC expressed less positive rate of hormone receptors and barely HER2, which was similar with SCC. ASC patients underwent the similar surgical and systematic treatment as IDC, only with less radiotherapy. Median follow-up data of 78 months showed that the prognosis of IDC patients was better than that of ASC patients (all *p* < 0.05 for BCSM and OS). ASC was not an independent prognosis factor of breast cancer. After propensity score matching (PSM), no significant difference in BCSM nor OS was observed between ASC and IDC groups. In HR-negative patients, the prognosis of ASC was similar with that of IDC, and both were superior to SCC. In HR-positive patients, the 5-year survival rate of ASC was 63.5%, which was far less than that in ASC of HR-negative (81.0%). Multivariate analysis showed that older age (age > 60) and advanced AJCC-stage were independent factors of poor prognosis in ASC, breast-conserving surgery was also ideally suited for ASC.

**Conclusions:**

ASC has unique clinicopathological characteristics and prognosis. It is imperative to focus on a more precise and personalized treatment management of ASC patients.

## Introduction

Adenosquamous carcinoma (ASC) is a rare and unique form of invasive mammary carcinoma that occurs less than 0.2% of all cases of breast cancer [1]. The earliest example of a breast tumor with adenosquamous features was reported in 1912 by Konjentzny. It was only in 1987, when Rosen and Ernsberger consolidated and described 11 such cases, that the term ‘ASC’ was established and entrenched [2]. Until now, in the World Health Organization (WHO) breast cancer classification (2019), ASC was characterized as a variant of metaplastic mammary carcinoma, and other subtypes of metaplastic breast cancer were fibromatosis-like metaplastic carcinoma, squamous cell carcinoma, spindle cell carcinoma, metaplastic breast cancer with mesenchymal differentiation [3].

Though several case reports and case series were published over the years [4, 5], ASC remains poorly defined by immunohistochemistry and its genetic profile is still unclear [6]. From these limited studies, it appears that ASC differs from its counterparts in this heterogeneous category by its relative clinical indolence, also reflects histologically in its cytomorphology [7]. Under normal conditions, squamous carcinoma (SCC) of the breast must be considered in the differential diagnosis as many reports have included tumors with varying proportions of squamous carcinoma and adenocarcinoma, very few are completely pure without other kind of glandular cell differentiation [8].

Currently, ASC has no consistent therapeutic strategy, the principles of treatment for ASC are either similar with those for SCC or for infiltrating duct carcinoma (IDC) according to the experience of doctors. However, accurate information concerning the comparison of breast ASC, breast SCC, and breast IDC has been unavailable.

In this way, the aim of our study is to perform a comparison of the prognosis among breast ASC, breast SCC, and breast IDC, and to further identify the underlying prognostic clinicopathological factors.

## Methods

### Data source and patient selection

The SEER program is a national database and a primary source of cancer statistics maintained by The National Cancer Institute. We have got permission to acquire the research data file in SEER*Stat Database: Incidence—SEER 18 Regs Custom Data (with additional treatment fields), Nov 2018 Sub (1975–2016 varying)—Linked To County Attributes—Total U.S., 1969–2017 Counties, National Cancer Institute, DCCPS, Surveillance Research Program, released April 2019, based on the April 2020 submission. The research data were obtained from 2004 to 2016 (Year of diagnosis = 2004–2016). We selected patients diagnosed with breast cancer (Site and Morphology. Site recode ICD-O-3/WHO 2008 = ‘Breast’) of infiltrating duct carcinoma, adenosquamous carcinoma, and squamous cell carcinoma based on International Classification of Diseases (ICD-O-3). The histological codes used to identify cases of infiltrating duct carcinoma and adenosquamous carcinoma were 8500/3 and 8560/3. The histological codes for retrieving squamous cell carcinoma were 8070/3, 8071/3, 8072/3, 8073/3, 8074/3, 8075/3, 8076/3, 8077/3, and 8078/3. Finally, a total of 557,203 patients were enrolled in our study, including 556,658 records of infiltrating duct carcinoma, contemporaneous 173 records of adenosquamous carcinoma, and 372 records of squamous cell carcinoma.

### Statistical analysis

The differences of demographic and clinicopathological features among IDC, ASC, and SCC groups were analyzed by the likelihood-ratio chi-squared test. Propensity Score Matching (PSM) method (Match Ratio 1:1; Logit model; the nearest neighbor matching approach) was used to eliminate demographic and clinic-pathological mixed bias in IDC and ASC groups. To estimate the PS score, we followed Dehejia [9] and Becker [10] and used the Logit model with the following steps. First, we started with estimating probabilities using the Logit model to select independent variables (list of variables in Table 1) which may affect the propensity of histology to be ASC. Then we calculated the propensity score (PS) which was the predicted values of the Logit model. The nearest neighbor matching method (PSMATCH2 [11]) was to search the closest control sample, both backwards and forwards, from the estimated PS values of the ASC group. After identifying the matching samples using nearest neighbor matching, we verified and compared (PSTEST [12, 13]) the extent of balancing between the two samples before and after having performed matching. Overall survival (OS) was defined as the time from admission to the date of death from any cause. For the breast cancer-specific mortality (BCSM), we included patients deceased from breast cancer and excluded patients who died from other causes. The OS curves and BCSM curves of each group were estimated by Kaplan–Meier survival analyses, and the curves were analyzed by the log-rank test. In the multivariate analysis, a COX’s Proportional Hazard Model was employed to estimate whether a factor was a significant independent prognostic factor of survival. All statistical tests were two-sided; *p* values less than 0.05 were considered as statistically significant. The statistical analyses were performed using STATA version 15.1 for Windows (StataCorp LLC).

**Table 1:** Comparisons of characteristics between IDC, ASC, and SCC of the breast

	IDC (*n* = 556,658)	ASC (*n* = 173)	SCC (*n* = 372)	*P*^b^
Age (years)^a^				
≤ 60	277,467 (49.85%)	75 (43.35%)	133 (35.75%)	0.001
> 60	279,191 (50.15%)	98 (56.65%)	239 (64.25%)	
Race				
Black	62,125 (11.23%)	29 (16.86%)	55 (14.86%)	0.001
White	440,619 (79.68%)	135 (78.49%)	296 (80.00%)	
Other^c^	50,275 (9.09%)	8 (4.65%)	19 (5.14%)	
Unknown	3639	1	2	
Marital status				
Married	301,735 (57.15%)	83 (50.61%)	155 (44.80%)	0.001
Unmarried^d^	226,212 (42.85%)	81 (49.39%)	191 (55.20%)	
Unknown	28,711	9	26	
Grade				
I	106,413 (19.97%)	72 (44.44%)	33 (10.61%)	0.001
II	222,624 (41.78%)	28 (17.28%)	91 (29.26%)	
III and UD^e^	203,762 (38.24%)	62 (38.27%)	187 (60.13%)	
Unknown	23,859	11	61	
T				
T1	332,600 (61.90%)	97 (58.43%)	64 (19.28%)	0.001
T2	156,472 (29.12%)	50 (30.12%)	130 (39.16%)	
T3	25,596 (4.76%)	12 (7.23%)	68 (20.48%)	
T4	22,656 (4.22%)	7 (4.22%)	70 (21.08%)	
Unknown	19,334	7	40	
*N*				
N0	369,171 (67.89%)	135 (79.88%)	243 (69.23%)	0.011
N1	127,280 (23.41%)	26 (15.38%)	72 (20.51%)	
N2	30,454 (5.60%)	5 (2.96%)	27 (7.69%)	
N3	16,880 (3.10%)	3 (1.78%)	9 (2.56%)	
Unknown	12,873	4	21	
M				
M0	521,034 (95.28%)	163 (95.32%)	311 (86.63%)	0.001
M1	25,805 (4.72%)	8 (4.68%)	48 (13.37%)	
Unknown	9819	2	13	
AJCC stage				
I	268,390 (50.47%)	85 (51.83%)	56 (16.82%)	0.001
II	183,336 (34.48%)	59 (35.98%)	158 (47.45%)	
III	57,101 (10.74%)	12 (7.32%)	75 (22.52%)	
IV	22,914 (4.31%)	8 (4.88%)	44 (13.21%)	
Unknown	24,917	9	39	
ER				
Negative	113,521 (21.24%)	112 (72.26%)	226 (78.47%)	0.001
Positive	421,059 (78.76%)	43 (27.74%)	62 (21.53%)	
Unknown	22,078	18	84	
PR				
Negative	169,969 (32.02%)	127 (81.94%)	251 (87.15%)	0.001
Positive	360,821 (67.98%)	28 (18.06%)	37 (12.85%)	
Unknown	25,868	18	84	
HER2				
Negative	248,135 (82.54%)	86 (95.56%)	135 (92.47%)	0.001
Positive	52,472 (17.46%)	4 (4.44%)	11 (7.53%)	
Unknown	256,051	83	226	
Subtype				
Luminal B	36,198 (12.06%)	3 (3.37%)	4 (2.74%)	0.001
Luminal A	209,788 (69.89%)	27 (30.34%)	40 (27.40%)	
HER2enriched	16,158 (5.38%)	1 (1.12%)	7 (4.79%)	
Triple Negative	38,011 (12.66%)	58 (65.17%)	95 (65.07%)	
Unknown	256,503	84	226	
Surgery				
No surgery	43,317 (7.81%)	9 (5.23%)	65 (17.66%)	0.001
BCS	292,986 (52.83%)	87 (50.58%)	111 (30.16%)	
Mastectomy	218,282 (39.36%)	76 (44.19%)	192 (52.17%)	
Unknown	2073	1	4	
Radiotherapy				
No/Unknown	287,579 (51.66%)	104 (60.12%)	254 (68.28%)	0.001
Yes	269,079 (48.34%)	69 (39.88%)	118 (31.72%)	
Chemotherapy				
No/Unknown	323,668 (58.14%)	109 (63.01%)	209 (56.18%)	0.319
Yes	232,990 (41.86%)	64 (36.99%)	163 (43.82%)	

*IDC* Infiltrating duct carcinoma, *ASC* Adenosquamous carcinoma, *SCC* Squamous cell carcinoma, *AJCC* American Joint Committee on Cancer, *ER* estrogen receptors, *PR* progesterone receptor, *BCS* breast-conserving surgery

^a^The median of age was 61

^b^*P* value of the likelihood-ratio chi-squared test

^c^Including American Indian/AK Native, Asian/Pacific Islander

^d^Including divorced, separated, single (never married), unmarried or domestic partner and widowed

^e^Including grade 3 and undifferentiated

## Results

### Differences of demographic and clinicopathological features among IDC, ASC, and SCC

After omitting censored data, an original of 557,203 female breast cancer patients were enrolled in our study. In total patients, 173 patients (3.10%) were diagnosed as adenosquamous carcinoma of breast (ASC group) and 372 patients (6.68%) were identified as squamous cell carcinoma (SCC group). The median of age was 61 in all patients. Age distribution was significantly different among the three groups, with a greater age among SCC participants and low proportion of patients over 60 years old in IDC groups. More patients of other race and higher proportion of married in IDC group did not constitute a meaningful result. Compared with IDC, ASC had similar tumor size but low histological grade and less lymph node metastasis, while SCC was just the opposite. More distant metastasis of SCC leads its advanced AJCC stage at the time of diagnosis. The molecular markers of ASC were close to SCC, such as less positive rate of hormone receptors (estrogen receptor (ER): ASC 27.74% and SCC 21.53%, progesterone receptor (PR): ASC 18.06% and SCC 12.85%), barely expression of HER2 (ASC 4.44% and SCC 7.53%), which were totally different to IDC (all *p* < 0.05). In terms of molecular subtype of breast cancer, triple-negative and Luminal A were more common in ASC due to the absence of HER2. ASC patients underwent the same treatment as IDC (chemotherapy 36.99% vs. 41.86%, breast conserving surgery (BCS) 50.58% vs. 52.83%, *p* > 0.05), only with less radiotherapy (39.88% vs. 48.34%, *p* < 0.05). The comparisons of features among the three groups were shown in Table 1.

### Survival analysis among IDC, ASC, and SCC patients

**Fig. 1:**
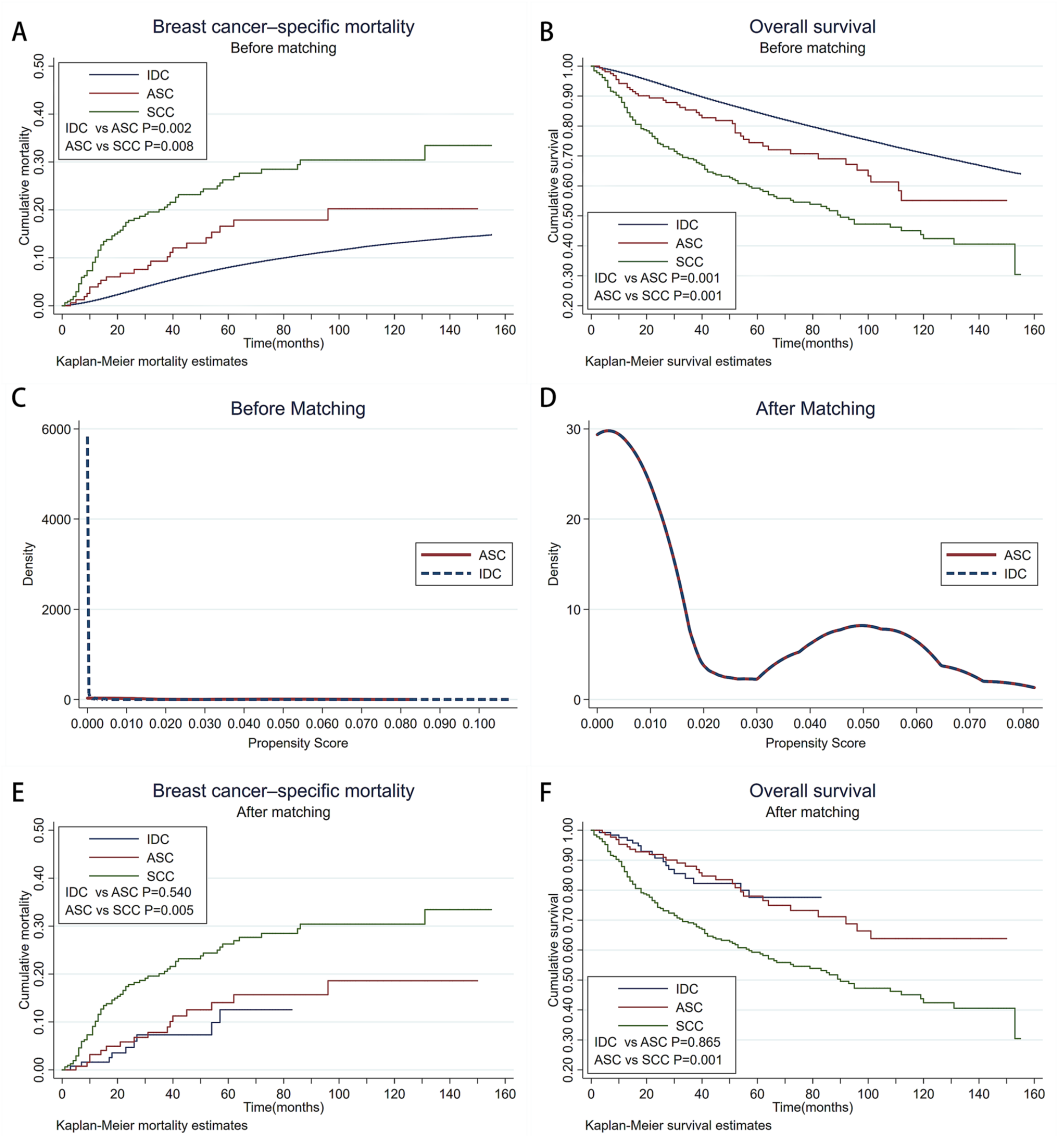
Kaplan–Meier curve illustrates BCSM and OS for IDC, ASC, and SCC in original and matched samples. **a** Kaplan–Meier curve illustrates BCSM for IDC, ASC, and SCC patients in original samples (IDC vs. ASC *p* = 0.002; ASC vs SCC *p* = 0.008, log-rank test); **b** Kaplan–Meier curve illustrates OS for IDC, ASC, and SCC patients in original samples (IDC vs. ASC *p* = 0.001; ASC vs. SCC *p* = 0.001, log-rank test); **c** Kernel Density of IDC and ASC groups before PS matching; **d** Kernel Density of IDC and ASC groups after PS matching. **e** Kaplan–Meier curve illustrates BCSM for IDC, ASC, and SCC patients in matched samples (IDC vs. ASC *p* = 0.540; ASC vs. SCC *p* = 0.005, log-rank test); **f** Kaplan–Meier curve illustrates OS for IDC, ASC and SCC patients in matched samples (IDC vs. ASC *p* = 0.865; ASC vs. SCC *p* = 0.001, log-rank test)

All breast cancer patients enrolled in our study were followed up for a median of 78 months (range of 1–155 months). After excluding the patients with distant metastases at the time of diagnosis (M1), we conducted survival analysis of BSCM and OS. By the end of the follow-up period, 92,278 IDC patients had died, 40,800 patients died of breast cancer, with the corresponding, 42 and 133 patients in ASC and SCC group had died, of which 22 and 72 patients due to recurrence and metastasis of breast cancer. In the total sample, the OS and BCSM of three histological group had noticeable difference from those of each other (OS: IDC vs. ASC *p* = 0.001; ASC vs. SCC *p* = 0.001, BCSM: IDC vs. ASC *p* = 0.002; ASC vs. SCC *p* = 0.008, log-rank test) (Fig. 1a, b).

### Survival analysis between IDC and ASC patients in PS matched groups

The propensity score matching method (Match Ratio 1:1; Logit model; the nearest neighbor matching approach) was employed to eliminate the bias of demographic and clinicopathological features between ASC and IDC groups (list of variables in Table 1). Because almost no expression of HER2 in ASC, we assumed the missing HER2 in ASC records before 2010 as negative to retain as many matched cases as possible. After matching, the hypothesis test showed that there was no statistical difference in the mean standard deviation and the standardized percentage bias of each variable between the two groups (Table 2). The kernel density functions showed that the general features between ASC group and IDC group (143 patients from the original ASC and IDC group, respectively) were similar (Fig. 1c, d). After PSM and omitting patients with distant metastases at initial diagnosis, 18 of 137 patients in IDC group had died, nine of whom owing to breast cancer. Accordingly, 16 patients died from breast cancer in 29 death cases of ASC. The OS and BCSM curve of ASC and IDC groups interwove with each other (*p* = 0.865 for OS and *p* = 0.540 for BCSM, log-rank test) (Fig. 1e, f). The prognosis of ASC seemed not inferior to that of IDC.

**Table 2:** Difference comparison of variables before and after PS matching (balancing assumption)

Variable	Unmatched	Mean		%bias	*t* test	
	Matched	ASC (*n* = 143)	IDC (*n* = 143)		*t*	*P*
Age	U	1.55	1.52	6.0	0.72	0.470
	M	1.55	1.57	− 5.6	− 0.47	0.635
Race	U	1.88	1.98	− 22.9	− 2.71	0.007
	M	1.88	1.94	− 12.4	− 0.97	0.332
Marital status	U	1.47	1.42	10.0	1.18	0.239
	M	1.47	1.53	− 10.5	− 0.85	0.394
*T*	U	1.62	1.51	13.1	1.61	0.107
	M	1.62	1.64	− 2.7	− 0.22	0.828
*N*	U	0.28	0.42	− 20.0	− 2.23	0.026
	M	0.28	0.38	− 14.0	− 1.14	0.256
*M*	U	0.04	0.04	0.6	0.07	0.946
	M	0.04	0.04	0.1	0.01	0.990
AJCC stage	U	1.67	1.67	0.4	0.05	0.961
	M	1.67	1.70	− 3.5	− 0.29	0.772
ER	U	0.27	0.81	− 127.9	− 6.39	0.001
	M	0.27	0.27	0.0	0.01	1
PR	U	0.20	0.71	− 119.8	− 13.46	0.001
	M	0.20	0.20	0.0	0.01	1
HER2	U	0.03	0.17	− 48.9	− 4.52	0.001
	M	0.03	0.03	0.0	0.01	1
Subtype	U	3.27	2.19	118.0	12.22	0.001
	M	3.27	3.30	− 3.5	− 0.23	0.815
Surgery	U	2.43	2.33	17.5	2.04	0.042
	M	2.43	2.43	0.0	0.01	1
Chemotherapy	U	0.41	0.51	− 18.7	− 2.22	0.026
	M	0.41	0.48	− 12.7	− 1.07	0.286
Radiotherapy	U	0.41	0.43	− 2.5	− 0.30	0.762
	M	0.41	0.41	0.0	0.01	1

### Clinical outcomes of IDC, ASC, and SCC in different breast cancer subtype groups

**Fig. 2:**
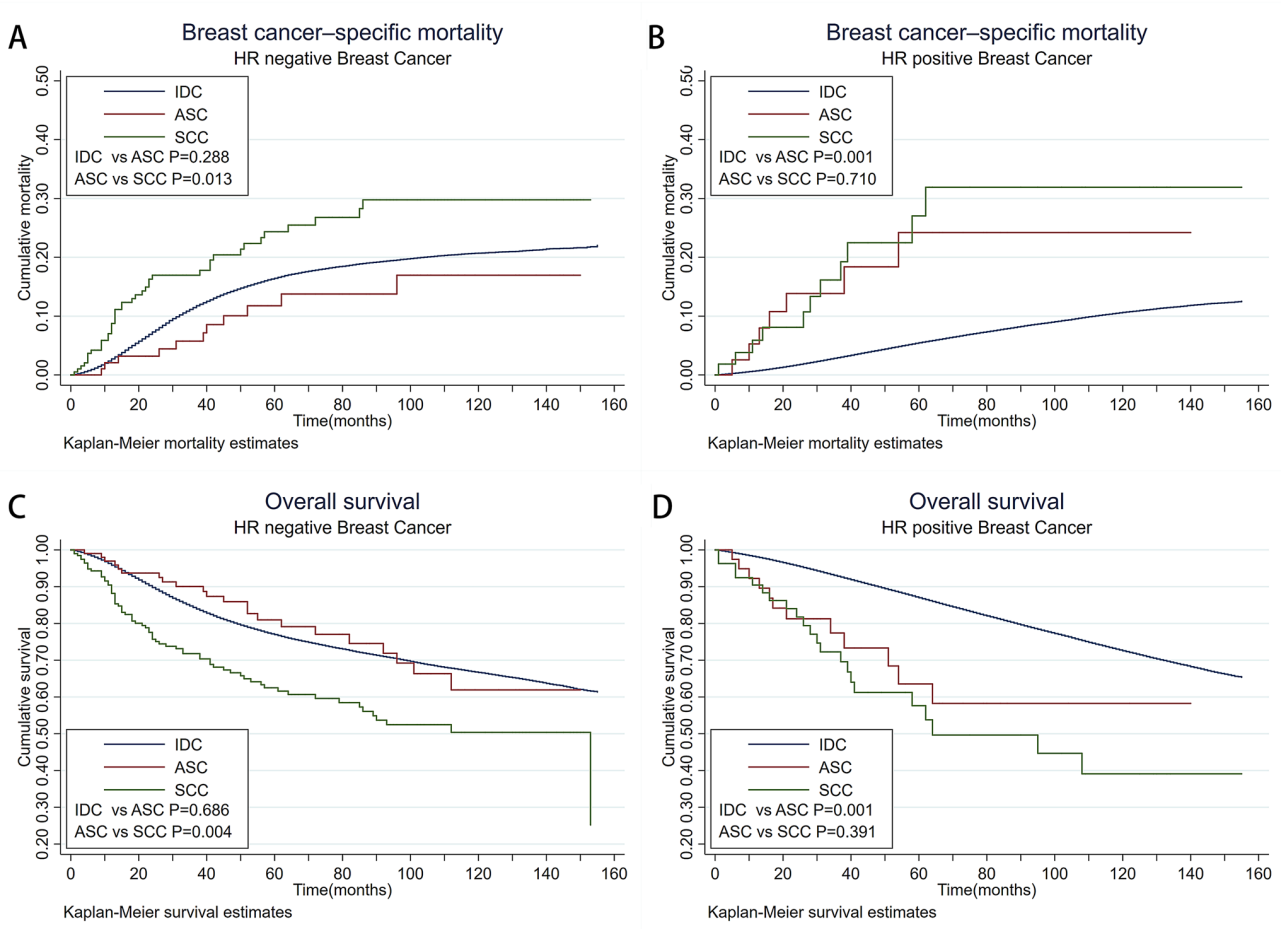
Kaplan–Meier curve illustrates BCSM and OS for IDC, ASC, and SCC in different HR subgroup. **a** Kaplan–Meier curve illustrates BCSM for IDC, ASC, and SCC in HR-negative subgroup (IDC vs. ASC *p* = 0.288; ASC vs. SCC *p* = 0.013, log-rank test); **b** Kaplan–Meier curve illustrates BCSM for IDC, ASC, and SCC in HR-positive subgroup (IDC vs. ASC *p* = 0.001; ASC vs. SCC *p* = 0.710, log-rank test); **c** Kaplan–Meier curve illustrates OS for IDC, ASC, and SCC in HR-negative subgroup (IDC vs. ASC *p* = 0.686; ASC vs. SCC *p* = 0.004, log-rank test); **d** Kaplan–Meier curve illustrates OS for IDC, ASC, and SCC in HR-positive subgroup (IDC vs. ASC *p* = 0.001; ASC vs. SCC *p* = 0.391, log-rank test)

Molecular subtypes of breast cancer play an essential role in guiding clinical treatment and predicting prognosis. In ASC group, the absence of HER2 expression led us to divide ASC into triple negative and luminal A only through hormone receptor expression. In HR-negative subgroup, we found that the OS and BCSM of ASC patients were close to that of IDC (*p* = 0.686 for OS and *p* = 0.288 for BCSM, log-rank test) (Fig. 2a, c). The prognosis of IDC and ASC with negative HR receptor was better than that of SCC with the same immunophenotype (all *p* < 0.05 for OS and BCSM between groups, log-rank test). On the contrary, in HR-positive subgroup, the prognosis of ASC was poor, which was similar to that of SCC (OS: IDC vs. ASC *p* = 0.001; ASC vs. SCC *p* = 0.391, BCSM: IDC vs ASC *p* = 0.001; ASC vs SCC *p* = 0.710, log-rank test) (Fig. 2b, d). The 5-year survival rate of ASC with HR-positive was 63.5%, which was far less than that in the HR-negative subgroup (81.0%) (Fig. 2c, d).

### Cox proportional hazards models for OS and BCSM

To further investigate the effect of baseline characteristics on prognosis of breast cancer, the multivariate Cox proportional hazards model was utilized to fitted for OS and BCSM. As shown in Table 3, as the consensus that had been achieved, demographic factors such as older age, black race, and unmarried were the poor prognostic factors for breast cancer, clinicopathological features such as higher histological grade, larger tumor size, more lymph node metastasis, and negative expression of HR/HER2 related to poor prognosis of breast cancer. Standard mastectomy/breast conserving surgery and adjuvant radiotherapy/chemotherapy brought survival benefits to the patients (all *p* < 0.05 for HR). However, after adjusting other prognostic factors, histology type of ASC was no longer an independent prognostic factor in multivariate analysis (HR = 1.07 for BCSM, 95% CI 0.40–2.84, *p* = 0.889; HR = 1.15 for OS, 95% CI 0.55–2.41, *p* = 0.716) (Table 3). SCC had poor prognosis in comparison with other two histological breast cancer (HR = 0.66 for OS, 95% CI 0.44–0.99, *p* = 0.044). We also analyzed the variables potentially influencing OS and BCSM of ASC by Cox proportional hazards model and Table 4 showed that only advanced AJCC stage (III) were independent factors of poor prognosis in ASC (*p* < 0.05 for HR). Elderly patients (age > 60) were associated with worse overall survival outcome in ASC patients (HR = 0.19 for OS, *p* = 0.003). BCS had the same therapeutic effect as mastectomy for OBC patients (HR = 2.34 for BCSM *p* = 0.069, HR = 0.96 for OS *p* = 0.924). Chemotherapy and radiotherapy also failed to bring significant survival benefits to ASC patients (all *p* > 0.05 for HR).

**Table 3:** Multivariate analyses of OS and BCSM using cox proportional hazards modeling

		BCSM		OS	
		HR (95%CI)	*P*	HR (95%CI)	*P*
Age (years)	> 60 vs. ≤ 60	1.43 (1.37–1.49)	0.001	0.44 (0.43–0.45)	0.001
Race	Black	Reference		Reference	
	White	0.81 (0.77–0.85)	0.001	1.15 (1.11–1.19)	0.001
	Other^a^	0.56 (0.52–0.62)	0.001	1.70 (1.60–1.82)	0.001
Marital status	Unmarried^b^ vs Married	1.28 (1.23–1.33)	0.001	0.66 (0.64–0.67)	0.001
Histology	IDC	Reference		Reference	
	ASC	1.07 (0.40–2.84)	0.889	1.15 (0.55–2.41)	0.716
	SCC	1.45 (0.86–2.46)	0.162	0.66 (0.44–0.99)	0.044
Grade	I	Reference		Reference	
	II	1.94 (1.76–2.14)	0.001	0.86 (0.82–0.89)	0.001
	III and UD^c^	3.54 (3.21–3.91)	0.001	0.61 (0.58–0.64)	0.001
*T*	T1	Reference		Reference	
	T2	2.16 (2.05–2.27)	0.001	0.57 (0.55–0.59)	0.001
	T3	3.37 (3.14–3.61)	0.001	0.40 (0.38–0.42)	0.001
	T4	4.90 (4.55–5.27)	0.001	0.28 (0.27–0.30)	0.001
*N*	N0	Reference		Reference	
	N1	1.86 (1.77–1.95)	0.001	0.71 (0.69–0.73)	0.001
	N2	3.30 (3.10–3.52)	0.001	0.42 (0.40–0.45)	0.001
	N3	4.78 (4.46–5.12)	0.001	0.30 (0.28–0.31)	0.001
ER	Positive vs. Negative	0.69 (0.65–0.73)	0.001	1.38 (1.32–1.44)	0.001
PR	Positive vs. Negative	0.55 (0.52–0.58)	0.001	1.45 (1.39–1.51)	0.001
HER2	Positive vs. Negative	0.65 (0.62–0.69)	0.001	1.28 (1.24–1.33)	0.001
Surgery	No surgery	Reference		Reference	
	BCS	0.26 (0.24–0.28)	0.001	3.02 (2.87–3.17)	0.001
	Mastectomy	0.32 (0.30–0.34	0.001	3.07 (2.94–3.21)	0.001
Chemotherapy	Yes vs. No/Unknown	0.69 (0.66–0.72)	0.001	1.79 (1.74–1.85)	0.001
Radiotherapy	Yes vs. No/Unknown	0.72 (0.69–0.76)	0.001	1.68 (1.63–1.73)	0.001

*BCSM* breast cancer-specific mortality, *OS* overall survival, *IDC* infiltrating duct carcinoma, *ASC* Adenosquamous carcinoma, *SCC* Squamous cell carcinoma, *HR* hazards ratio, *CI* confidence interval, *ER* estrogen receptors, *PR* progesterone receptor, *BCS* breast-conserving surgery

^a^Including American Indian/AK Native, Asian/Pacific Islander

^b^Including divorced, separated, single (never married), unmarried or domestic partner and widowed

^c^Including grade 3 and undifferentiated

**Table 4:** Factors affecting BCSM and OS in patients with Adenosquamous Carcinoma

	Univariate analyses^a^		Multivariate analyses	
	BCSM		OS	
	HR (95%CI)	*P*	HR (95%CI)	*P*
Age (years)				
≤ 60	Reference		Reference	
> 60	2.15 (0.84–5.51)	0.109	0.19 (0.06–0.56)	0.003
Grade				
I + II	Reference		Reference	
III and UD^b^	2.33 (0.98–5.53)	0.055	0.44 (0.16–1.19)	0.105
AJCC stage				
I + II	Reference		Reference	
III	8.43 (3.13–22.68)	0.001	0.12 (0.03–0.40)	0.001
Subtype				
Luminal	Reference		Reference	
Non-Luminal	2.03 (0.78–5.27)	0.145	0.71 (0.28–1.80)	0.469
Surgery				
BCS	Reference		Reference	
Mastectomy	2.34 (0.94–5.89)	0.069	0.96 (0.37–2.44)	0.924
Radiotherapy				
No/Unknown	Reference		Reference	
Yes	0.71 (0.29–1.73)	0.448	0.76 (0.32–1.84)	0.545
Chemotherapy				
No/Unknown	Reference		Reference	
Yes	1.45 (0.63–3.36)	0.385	1.02 (0.38–2.74)	0.977

*BCSM* breast cancer-specific mortality, *OS* overall survival, *HR* hazards ratio, *CI* confidence interval, *BCS* breast-conserving surgery

^a^22 *breast cancer-specific deaths* occurred

^b^Including grade 3 and undifferentiated

## Discussion

Most studies of ASC of the breast had been small series or single case reports because of its rarity [14]. Therefore, clinicopathological features and outcomes of this entity remained unclear. In the present study, we described clinical characteristics of patients with ASC of the breast and identified variables affecting BCSM and OS using data from SEER. Only 173 patients recorded in SEER diagnosed as ASC between 2004 and 2016 were extracted from the database. Compared with 556,658 cases with IDC of the breast contemporaneously, the prevalence of ASC of the breast was very low.

According to our results, median age at diagnosis of patients was 61 years, and higher proportion of ASC patients older than the median age meant ASC was more commonly found in middle-aged and older female. In this cohort, white patients accounted for the largest proportion (~ 78.49%), which was consistent with the distribution of races in the Western population.

In our study, ASC patients had lower histological grade and less lymph node metastasis than IDC patients, however, after matching, these characteristics did not give them better survival outcomes than IDC patients. On the contrast, though SCC patients had similar tumor size, histological grade and lymph node metastasis to IDC patients, they came up with the worst survival outcomes among these three histological types of breast cancer. Compared with ASC in other site, breast ASC patients predicted favorable prognosis. For instance, the prognosis of gastric ASC was worse than that of gastric adenocarcinoma [15]. Besides, lung ASC had higher grade malignancy, stronger lymph nodal invasiveness, more frequent brain metastases and poorer prognosis than lung adenocarcinoma and SCC [16]. However, in other studies, there were also ASC in some site behaved similar with common type. As an example, patients with gallbladder ASC were similar to those with adenocarcinoma of the gallbladder in clinical characteristics and features, although the ASC patients were more prone to infiltration of multiple adjacent organs and lymphatic metastasis [17]. In addition, esophageal ASC behaves more like adenocarcinoma in response to chemoradiotherapy and survival based on treatment modality [18]. Under these circumstances, we recommended clinical doctors not evaluating prognosis of ASC patients only by tumor size, histological grade or lymph node metastasis.

Then we focused our attention on molecular markers, nearly all ASC patients were HER2 negative, which gave an explanation to why Luminal A and TNBC accounted for larger proportion of all molecular classifications. To our surprise, ER/PR expression in ASC patients seemed more like that in SSC patients rather than that in IDC patients. Since ASC and SCC were partly similar in pathology, they were both positive in cytokeratin 5/6 (CK5/6) [8], cytokeratin 10/13 (CK10/13) or p63 [5, 8]; however, there was much distance between survival outcomes of ASC patients and SCC patients. In that case, we recommended clinical doctors noticing pathological differentiate diagnosis.

Besides, we found that different molecular classifications could exert a profound influence on survival prognosis of ASC patients. Five-year survival rate of ASC subgroup with hormone receptor positive was far less than that of the HR-negative subgroup, this result was contrary to IDC patients. There was a case report suggesting that when the expression of hormone receptors was positive in ASC, CD44v could play an important role in the transition of LGASC precursor lesions into malignant processes [7]. CD44v, a widely accepted cancer stem cell (CSC) marker in breast cancer, was considered to promote the tumor progression in various cancers [19]. However, there is no statistical difference between Luminal and Non-luminal in the multivariate analysis, this could have a better explanation, which required a deeper study with larger samples.

We found that most ASC patients received surgery (~ 94.18%), with a BCS to mastectomy ratio of 1.14:1. Besides, they also received radiotherapy (~ 42.31%) and chemotherapy (~ 34.62%). Probably due to lack of understanding of ASC, half doctors still chose mastectomy rather than BCS, combined with the exclusion of multiple factors offset cox, mastectomy did not lead to a better survival prognosis than BCS, so BCS was still the appropriate choice for ASC under the reasonable indications.

## Conclusion

The present study has shown patients with ASC of the breast to be not exactly the same as those with IDC of the breast in clinical characteristics and features. Although the ASC patients were less prone to lymphatic metastasis, the prognosis of ASC was similar to that of IDC. Molecular markers may play an important role in dividing ASC patients into better or worse prognosis groups. Both BCS and mastectomy can effectively improve the prognosis of these patients. Further studies with larger sample sizes from multiple institutions are needed to confirm clinicopathological features and survival rates of ASC.

## Data Availability

All data generated or analyzed during this study are included in this published article [and its supplementary information files].

## Abbreviations

ASC: Adenosquamous carcinoma
BCS: Breast-conserving surgery
BCSM: Breast cancer-specific mortality
CI: Confidence interval
ER: Estrogen receptor
HER2: Human epidermal growth factor receptor 2
HR: Hazard ratio
IDC: Infiltrating duct carcinoma
OS: Overall survival
PSM: Propensity score matching
PR: Progesterone receptor
SCC: Squamous carcinoma
SEER: Surveillance, Epidemiology, and End-Results database
WHO: World Health Organization
